# Association of *RET* Genetic Polymorphisms and Haplotypes with Papillary Thyroid Carcinoma in the Portuguese Population: A Case-Control Study

**DOI:** 10.1371/journal.pone.0109822

**Published:** 2014-10-17

**Authors:** Marina Santos, Teresa Azevedo, Teresa Martins, Fernando J. Rodrigues, Manuel C. Lemos

**Affiliations:** 1 CICS-UBI, Health Sciences Research Centre, Faculty of Health Sciences, University of Beira Interior, Covilhã, Portugal; 2 Endocrinology Service, Portuguese Institute of Oncology, Coimbra, Portugal; IPATIMUP/Faculty of Medicine of the University of Porto, Portugal

## Abstract

Thyroid cancer has a multifactorial aetiology resulting from the interaction of genetic and environmental factors. Several low penetrance susceptibility genes have been identified but their effects often vary between different populations. Somatic point mutations and translocations of the REarranged during Transfection (*RET*) proto-oncogene are frequently found in thyroid cancer. The aim of this case-control study was to determine the effect of four well known *RET* single nucleotide polymorphisms (SNPs) on the risk for differentiated thyroid carcinoma. A total of 545 Portuguese patients and 543 controls were genotyped by PCR and restriction enzyme analysis, for the following SNPs: G691S (exon 11, rs1799939 G/A), L769L (exon 13, rs1800861 T/G), S836S (exon 14, rs1800862 C/T), and S904S (exon 15, rs1800863 C/G). The minor allele of S836S was overrepresented in patients with papillary thyroid carcinoma (PTC) when compared to controls (OR 1.57; 95% CI 1.05–2.35; p = 0.026). The GGTC haplotype was also overrepresented in PTC (OR 2.51; 95% CI 1.07–5.91; p = 0.029). No associations were found in follicular thyroid carcinoma (FTC). Multivariate logistic regression analysis showed no differences regarding gender, age at diagnosis, lymph node or distant metastasis. However, a near significant overrepresentation of the minor alleles of G691S and S904S was found in patients with tumours greater than 10 mm of diameter at diagnosis. These data suggest that the *RET* S836S polymorphism in exon 14 and the GGTC haplotype are risk factors for PTC, but not FTC, and that the G691S/S904S polymorphisms might be associated with tumour behaviour.

## Introduction

Thyroid cancer is the most common malignancy of the endocrine system. The two most common forms of thyroid cancer are papillary thyroid carcinoma (PTC) and follicular thyroid carcinoma (FTC). These are collectively designated as differentiated thyroid carcinomas and are derived from thyroid follicular cells. Other less common forms are medullary thyroid carcinoma (MTC) which derives from thyroid parafollicular cells and anaplastic thyroid carcinoma which is a dedifferentiated highly aggressive cancer. Thyroid cancer has a strong hereditability, and individual susceptibility to thyroid cancer is likely to be modulated by the combination between the environment and multiple low- to moderate-penetrance genes interacting with each other [1], [2]. Exposure to ionizing radiation is the only established environmental factor related to thyroid cancer [3].

The human REarranged during Transfection (*RET*) proto-oncogene, located on chromosome 10q11.2, encodes a transmembrane receptor of the tyrosine-kinase family of proteins that is associated with a variety of disorders [4]. Loss-of-function mutations of the *RET* proto-oncogene results in Hirschsprung disease, a genetic disorder characterized by congenital absence of enteric neurons in the gastrointestinal tract. In contrast, gain-of-function mutations leading to aberrant RET activation, are involved in a number of human cancers [5]. Germline point mutations of *RET* are responsible for the development of heritable forms of MTC, while somatic mutations of *RET* are found in sporadic MTCs [6]. Furthermore, somatic rearrangements of the *RET* gene are associated with PTC and commonly seen in childhood tumours and those associated with radiation exposure [7].

The *RET* gene has several polymorphisms, but four have been more extensively studied as they occur in exons where most germline mutations in MTC are concentrated [8]. These *RET* polymorphisms, G691S (exon 11, rs1799939), L769L (exon 13, rs1800861), S836S (exon 14, rs1800862), and S904S (exon 15, rs1800863), have been repeatedly implicated in the increase of the risk of MTC [9], [10]. However, the effect of these single nucleotide polymorphisms (SNPs) on other types of thyroid cancer remains to be determined. Until now, only four studies investigated *RET* polymorphisms in non-MTCs, but these were relatively small and produced conflicting results [11], [12], [13], [14].

The aim of this study was to determine the contribution of the G691S, L769L, S836S and S904S *RET* polymorphisms to the genetic susceptibility to differentiated thyroid carcinoma in the Portuguese population.

## Materials and Methods

### Subjects

The study was designed as a retrospective case-control association study. Cases consisted of 545 Caucasian Portuguese patients with thyroid cancer (98 males and 447 females; mean age ± SD = 47.4±15.1 years) who attended the outpatient clinics at the Portuguese Institute of Oncology, Coimbra (Portugal). Patients were selected on the basis of histologically confirmed presence of any of the two major subtypes of differentiated thyroid carcinoma, i.e. papillary (n = 474) and follicular (n = 67) thyroid carcinoma. The control group consisted of 543 (208 males and 335 females; mean age ± SD = 30.0±12.6 years) Caucasian Portuguese unrelated volunteers who were recruited among blood donors from the same geographical area. Written informed consent was obtained from patients and controls and the study was approved by the local research ethics committees (Portuguese Institute of Oncology of Coimbra, and Faculty of Health Sciences, University of Beira Interior, Ref: CE-FCS-2012-011).

### Genetic studies

Genomic DNA was extracted from peripheral blood leucocytes using previously described methods [15]. Exons 11, 13, 14 and 15 of the *RET* gene were amplified by polymerase chain reaction (PCR) using previously described primer sequences [16] and optimized PCR conditions. Amplified fragments were digested with the appropriate restriction enzyme (New England Biolabs, Beverly, MA, USA) according to the manufacturer’s instructions and visualized on a 3% agarose gel.

The G691S (exon 11, rs1799939 G/A) SNP was analyzed by digestion of a 562-base pair (bp) PCR product with *Ban*I, which resulted in four fragments (17, 147, 175 and 223 bp) in the presence of the G allele and in three fragments (17, 147, 398 bp) in the presence of the A allele. The L769L (exon 13, rs1800861 T/G) SNP was analyzed by digestion of a 348-bp PCR product with *Taq*I, which resulted in two fragments (164, 184 bp) in the presence of the T allele and one uncut fragment (348 bp) in the presence of the G allele. The S836S (exon 14, rs1800862 C/T) SNP was analyzed by digestion of a 549-bp PCR product with *Alu*I, which resulted in five fragments (32, 42, 107, 158, 210 bp) in the presence of the C allele and four fragments (32, 42, 107, 368 bp) in the presence of the T allele. The S904S (exon 15, rs1800863 C/G) SNP was analyzed by digestion of a 358-bp PCR product with *Rsa*I, which resulted in one uncut fragment (358 bp) in the presence of the C allele and two fragments (123, 235, bp) in the presence of the G allele.

### Statistical analysis

The allelic and genotypic frequencies for each polymorphism in patients and controls were determined by direct counting. *RET* haplotypes derived from the four SNPs were reconstructed using Haploview software (version 4.2) [17]. Genotype, allele and haplotype frequencies in patients (PTC and FTC) and controls were compared by Pearson’s chi-square test of independence and the corresponding odds ratios (OR) with 95% confidence intervals (CI) were determined using SPSS 20.0 software (SPSS Inc., Chicago, IL, USA). Statistical significance was set at p<0.05. The best model of inheritance for each SNP (co-dominant, dominant, recessive, overdominant or additive) was selected using Akaike information criterion (AIC) and SNPStats software [18].

Subgroup analysis was carried out by multivariate logistic regression to assess the effect of *RET* polymorphisms according to gender (male or female), age at diagnosis (<45 or ≥45 years), tumour size at diagnosis (<10 or ≥10 mm of diameter) and presence of lymph node or distant metastasis. The results of multivariate logistic regression were corrected for multiple testing using the Bonferroni and False Discovery Rate (FDR) corrections [19]. Correction for the number of SNPs was not performed as the four studied SNPs were in tight linkage disequilibrium, and therefore were not independent variables.

Hardy-Weinberg equilibriums were assessed using the chi-squared goodness-of-fit test to compare the observed and allele-based expected genotype frequencies. The linkage disequilibrium (LD) coefficients, D′ and r2, were assessed by Haploview software (Version 4.2) [17]. Power calculation was analyzed using the program Power and Sample Size Calculations (Version 3.0) [20]. Assuming a minor allele frequency of 20%, it was estimated that the study sample was sufficient to detect an OR of 1.5 for PTC and an OR of 2.2 for FTC, with an estimated power of 0.8 and a type 1 error probability of 0.05.

## Results

The *RET* genotype, allele and haplotype frequencies observed in patients and controls are presented in Table 1. Genotypes were in Hardy-Weinberg equilibrium. The G691S and S904S SNPs were in complete linkage disequilibrium (D' = 1.0; r2 = 0.99). No differences were found in the distribution of genotypes, alleles and haplotypes between male and female controls, or between the younger (mean age ± SD, 18.9±0.9 years) and older (mean age ± SD, 48.8±9.6 years) quartiles, indicating that differences in the sex ratio and mean age between the groups were unlikely to influence the results.

**Table 1:** *RET* genotype, allele and haplotype frequencies in patients and controls.

		Controls	All patients		PTC		FTC	
Polymorphism		total = 543	total = 545	OR (95% CI)	total = 474	OR (95% CI)	total = 67	OR (95% CI)
		n (%)	n (%)		n (%)		n (%)	
G691S genotype	GG	355 (65.4)	340 (62.4)	0.88 (0.69–1.13)	295 (62.2)	0.87 (0.68–1.13)	42 (62.7)	0.89 (0.53–1.51)
	GA	169 (31.1)	183 (33.6)	1.12 (0.87–1.44)	161 (34.0)	1.14 (0.88–1.48)	21 (31.3)	1.01 (0.58–1.75)
	AA	19 (3.5)	22 (4.0)	1.16 (0.62–2.17)	18 (3.8)	1.09 (0.56–2.10)	4 (6.0)	1.75 (0.58–5.31)
G691S allele	G	879 (80.9)	863 (79.2)	0.90 (0.73–1.11)	751 (79.2)	0.90 (0.72–1.12)	105 (78.4)	0.85 (0.55–1.32)
	A	207 (19.1)	227 (20.8)	1.12 (0.90–1.38)	197 (20.8)	1.11 (0.90–1.39)	29 (21.6)	1.17 (0.76–1.82)
L769L genotype	TT	331 (61.0)	330 (60.6)	0.98 (0.77–1.25)	295 (62.2)	1.06 (0.82–1.36)	34 (50.7)	0.66 (0.40–1.10)
	TG	192(35.4)	187 (34.3)	0.95 (0.74–1.23)	154 (32.5)	0.88 (0.68–1.14)	30 (44.8)	1.48 (0.89–2.48)
	GG	20 (3.7)	28 (5.1)	1.42 (0.79–2.55)	25 (5.3)	1.46 (0.80–2.66)	3 (4.5)	1.23 (0.35–4.24)
L769L allele	T	854 (78.6)	847 (77.7)	0.95 (0.77–1.16)	744 (78.5)	0.99 (0.80–1.23)	98 (73.1)	0.74 (0.49–1.11)
	G	232 (21.4)	243 (22.3)	1.06 (0.86–1.29)	204 (21.5)	1.01 (0.82–1.25)	36 (26.9)	1.35 (0.90–2.03)
S836S genotype	CC	500 (92.1)	480 (88.1)	0.64 (0.42–0.95)	416 (87.8)	0.62 (0.41–0.93)	60 (89.6)	0.74 (0.32–1.71)
	CT	42 (7.7)	64 (11.7)	**1.59 (1.05–2.39) ^(b)^**	57 (12.0)	**1.63 (1.07–2.48) ^(c)^**	7 (10.4)	1.39 (0.60–3.24)
	TT	1 (0.2)	1 (0.2)	1.00 (0.06–15.97)	1 (0.2)	1.15 (0.07–18.37)	0 (0.0)	–
S836S allele	C	1042 (95.9)	1024 (93.9)	0.66 (0.44–0.97)	889 (93.8)	0.64 (0.43–0.95)	127 (94.8)	0.77 (0.34–1.74)
	T	44 (4.1)	66 (6.1)	**1.53 (1.03–2.26) ^(d)^**	59 (6.2)	**1.57 (1.05–2.35) ^(e)^**	7 (5.2)	1.31 (0.58–2.96)
S904S genotype	CC	354 (65.2)	339 (62.2)	0.88 (0.69–1.13)	294 (62.0)	0.87 (0.68–1.13)	42 (62.7)	0.90 (0.53–1.52)
	CG	170 (31.3)	184 (33.8)	1.12 (0.87–1.44)	162 (34.2)	1.14 (0.88–1.48)	21 (31.3)	1.00 (0.58–1.73)
	GG	19 (3.5)	22 (4.0)	1.16 (0.62–2.17)	18 (3.8)	1.09 (0.56–2.10)	4 (6.0)	1.75 (0.58–5.31)
S904S allele	C	878 (80.8)	862 (79.1)	0.90 (0.73–1.11)	750 (79.1)	0.90 (0.72–1.12)	105 (78.4)	0.86 (0.55–1.33)
	G	208 (19.2)	228 (20.9)	1.12 (0.90–1.38)	198 (20.9)	1.11 (0.90–1.39)	29 (21.6)	1.17 (0.75–1.81)
Haplotype ^(a)^		total = 1086	total = 1090		total = 948		total = 134	
		n (%)	n (%)		n (%)		n (%)	
G/T/C/C		642 (59.1)	611 (56.1)	0.88 (0.74–1.05)	538 (56.8)	0.91 (0.76–1.08)	71 (53.0)	0.78 (0.54–1.12)
A/T/C/G		205 (18.9)	227 (20.8)	1.13 (0.92–1.40)	197 (20.8)	1.13 (0.91–1.40)	27 (20.2)	1.08 (0.69–1.70)
G/G/C/C		192 (17.7)	185 (17.0)	0.95 (0.76–1.19)	153 (16.1)	0.90 (0.71–1.13)	29 (21.6)	1.29 (0.83–2.00)
G/G/T/C		38 (3.5)	58 (5.3)	**1.55 (1.02–2.35) ^(f)^**	51 (5.4)	**1.57 (1.02–2.41) ^(g)^**	5 (3.7)	1.07 (0.41–2.76)

PTC, papillary thyroid carcinoma; FTC, follicular thyroid carcinoma; n, number; %, percentage; OR, odds ratio; CI, confidence interval; (a), G691S/L769L/S836S/S904S (only haplotypes with frequencies >1% are presented); (b), p = 0.026 (overdominant inheritance model); (c), p = 0.021 (overdominant inheritance model); (d), p = 0.033 (additive inheritance model); (e), p = 0.026 (additive inheritance model); (f), p = 0.039; (g), p = 0.039.

The frequency of the S836S minor allele (T allele) was higher in patients with thyroid cancer compared to controls (OR 1.53; 95% CI 1.03–2.26; p = 0.033). When analysed according to cancer subtype, this increased frequency was observed in patients with PTC (OR 1.57; 95% CI 1.05–2.35; p = 0.026), but not in patients with FTC. The increase of the minor allele frequency was accompanied by an increase of the frequency of the heterozygous CT genotype, under an overdominant inheritance model (Table 1).

The GGTC haplotype was also overrepresented in PTC (OR 2.51; 95% CI 1.07–5.91; p = 0.029) (Table 1). Multivariate logistic regression analysis showed no differences regarding gender, age at diagnosis, lymph node or distant metastasis (data not shown). The minor alleles of G691S (OR = 1.624; 95% CI = 1.06–2.48; p = 0.025) and S904S (OR = 1.57; 95% CI = 1.03–2.39; p = 0.038) were associated with tumour size at diagnosis ≥10 mm of diameter (n = 353) compared to tumour size <10 mm (n = 146). However, after correcting for multiple comparisons using both the Bonferroni and False Discovery Rate (FDR) corrections, this association with tumour size no longer remained significant (Bonferroni p = 0.100 and p = 0.152, FDR p = 0.084 and p = 0.128, respectively).

## Discussion

This case-control study revealed an increased frequency of the *RET* S836S minor allele (T allele) in patients with PTC when compared to controls. This is similar to that observed in the study of Ho et al. [12] in a North American population. However, the later study was relatively small (101 patients), borderline significant (p = 0.051), and did not discriminate between the different types of differentiated thyroid cancer. Another study by Sigurdson et al. [14] in a radiation-exposed population from Kazakhstan, showed that the S836S minor allele was overrepresented in patients with thyroid nodules, and possibly with PTC, although the number of PTC patients was small (n = 25). Two other studies did not show this association [11], [13]. The studies by Lesueur et al. [11], on a mixed European/Australian population (247 patients), and by Lonn et al. [13], on a North American population (167 patients), did not show an association with the S836S SNP, but showed associations with the L769L and G691S SNPs, respectively.

Our study is the largest reported so far, on the association between differentiated thyroid cancer and polymorphisms at the *RET* locus. The results suggest that the S836S polymorphism in exon 14 is a risk factor for PTC, but not for FTC. The different results for these two cancer subtypes may reflect the smaller number of FTC patients, which reduced the power of the analysis, or may reflect the differences in the aetiology of these tumours, since alterations of the RET signalling pathway are commonly found in PTC, but not FTC [7]. The results also suggest that the GGTC haplotype is associated with an increased risk for PTC. This haplotype includes the T allele of the S836S polymorphism that is associated with the increased risk in the single locus analysis, but it also includes the minor allele of the L769L polymorphism. The combination of these two minor alleles may cause a synergistic effect and may amplify the effects of the single polymorphisms, as shown for other tumours [21]. In addition, multivariate analysis of tumour parameters showed a near significant association between the minor alleles of G691S/S904S polymorphisms and larger tumour size (≥10 mm). This size cutoff was chosen since microcarcinomas (defined as size smaller than 10 mm) are usually associated with a lack of clinical significance and are mostly diagnosed as post-operative incidental findings [22]. These findings suggest that the G691S/S904S polymorphisms may increase the risk for more aggressive disease and that future association studies should take into account the size of the tumour.

The mechanisms by which the *RET* polymorphisms may confer an increased susceptibility to PTC remain to be understood. The G691S polymorphism involves a change of amino acid in the RET protein sequence (glycine to serine) and may have a subtle effect on its function [9], [23]. However, the other three studied polymorphisms are silent and do not lead to amino acid changes, so the mechanism by which these SNPs may act to increase the risk for disease remains to be identified. The possibility that these SNPs influence RNA stability or protein synthesis [24], or that they are in linkage disequilibrium with another unknown functional nucleotide variant, cannot presently be excluded. Interestingly, the S836S minor allele (T allele) which was found to be associated with PTC in our study, was previously found to be over-represented in patients with MTC [10] and under-represented in patients with Hirschsprung disease [25], and these disorders are known to be caused by RET overactivity and underactivity, respectively. This suggests a non-neutral effect of this polymorphism on RET function.

The results of this study should be viewed with caution as the risk associated with the studied SNPs is only marginally significant, with ORs between 1.5 and 2.5, and p-values between 0.01 and 0.05. In addition, any existing effect of these SNPs may be population-specific, depending on the genetic background and environmental exposure of the studied populations. In particular, exposure to radiation, which is a known physical carcinogen for PTC, was not evaluated in this study.

In summary, our data suggest that the *RET* S836S polymorphism in exon 14 and the GGTC haplotype are risk factors for PTC and that the G691S/S904S polymorphisms might be associated with tumour behaviour. Further studies with larger populations, including cases of FTC, may prove useful to assess the role of *RET* polymorphisms in differentiated thyroid cancer.
